# Responsible AI measures dataset for ethics evaluation of AI systems

**DOI:** 10.1038/s41597-025-06021-5

**Published:** 2025-12-20

**Authors:** Shalaleh Rismani, Leah Davis, Bonam Mingole, Negar Rostamzadeh, Renee Shelby, AJung Moon

**Affiliations:** 1 McGill University, Montréal, Canada; 2 Pennsylvania State University, State College, USA; 3 Google Research, Montreal, Canada; 4 Google Research, San Francisco, USA

**Keywords:** Technology, Industry, Ethics

## Abstract

Meaningful governance of any system requires the system to be assessed and monitored effectively. In the domain of Artificial Intelligence (AI), global efforts have established a set of ethical principles, including fairness, transparency, and privacy upon which AI governance expectations are being built. The computing research community has proposed numerous means of measuring an AI system’s normative qualities along these principles. Current reporting of these measures is principle-specific, limited in scope, or otherwise dispersed across publication platforms, hindering the domain’s ability to critique its practices. To address this, we introduce the Responsible AI Measures Dataset, consolidating 12,067 data points across 791 evaluation measures covering 11 ethical principles. It is extracted from a corpus of computing literature (*n* = 257) published between 2011 and 2023. The dataset includes detailed descriptions of each measure, AI system characteristics, and publication metadata. An accompanying, interactive visualization tool supports usability and interpretation of the dataset. The Responsible AI Measures Dataset enables practitioners to explore existing assessment approaches and critically analyze how the computing domain measures normative concepts.

## Background & Summary

The development and governance of technology require the integration of holistic measurement frameworks to provide essential feedback on system quality, safety, and performance, enabling developers and policymakers to identify and mitigate potential harms. For instance, in the automotive industry, vehicles must meet specific performance benchmarks (e.g., achieving certain speed thresholds), safety standards (e.g., minimizing driver impact during a head-on collision), and environmental requirements (e.g., limiting exhaust emissions) before they are put on the market. Governance expectations for Artificial Intelligence (AI) systems should be no exception. With the integration of AI into products and services, it is necessary to evaluate their inherent risks beyond basic performance metrics, such as accuracy^1–3^. Ethical principles are increasingly emphasized as guiding constructs for AI system assessments; current and upcoming regulations underscore the necessity for AI systems to be evaluated on factors such as fairness, transparency, security, privacy, and sustainability^4,5^.

Just as governments and organizations measure abstract concepts like economic health (e.g., GDP, employment rates) or social inequality (e.g., gender wage gap, housing affordability)^6^, the AI community has sought ways to quantify normative concepts such as fairness, accountability, and transparency^7^. For example, the *Responsible AI Index* (https://www.global-index.ai/) ranks countries based on their progress toward ethical AI adoption, offering a valuable macro-level assessment of responsible AI benchmarks^8^. While high-level initiatives are useful for guiding policy and societal awareness, practitioners require more granular measures to evaluate AI systems against these principles in practice^5^. Consequently, there is momentum within the computing research community to develop measures that practitioners can use to evaluate how AI systems uphold these principles^9–11^. These measures are essential for closing the feedback loop, enabling developers and organizations to identify normative issues and establish harm-reductive governance practices.

Despite various efforts to synthesize existing measures, several challenges remain. Many existing reviews focus on measures related only to a single principle (e.g., fairness or transparency)^10,11^ or a specific type of AI system (e.g., computer vision or recommendation systems)^9,12^, overlooking relationships between measures across principles, system types, or assessment types and contexts. This fragmentation underscores the need for a comprehensive database that addresses these challenges by examining how measures are reported and documented across principles, identifying relationships between them, and clarifying their application to specific components of an AI system as defined by the Organisation for Economic Co-operation and Development (OECD)^13^. Such a database should also encompass a diverse range of assessment types–mathematical, statistical, behavioural, and self-reported–while ensuring consistency in terminology.

The demand for more comprehensive synthesis is further underscored by mounting criticism from computing^14,15^, human-computer interaction (HCI)^16^, and computational social science communities^17^ regarding the limitations of current measurement practices for responsible AI development. Scholars highlight that many widely used measures lack construct reliability and validity, undermining their effectiveness^15^. Jacobs and Wallach, for example, argue that fairness measures often fail to define the constructs they aim to assess, resulting in unreliable and invalid results^17^. Extending this critique, Blodgett *et al*. examine benchmarking datasets used for detecting stereotypes and demonstrate how poor construct validity compromises their applicability^14^. To address these criticisms, a number of studies have explored measurement modelling–based approaches; among them, Xiao *et al*. propose integrating measurement modelling concepts to assess the reliability and validity of natural language generation (NLG) metrics^18^.

Beyond measurement modelling, critiques also focus on the sociotechnical gap– a concept introduced by Ackerman^19^ that underscores the misalignment between technical evaluations and the social contexts in which AI systems operate. Liao *et al*. argue that evaluations of large language models (LLMs) often lack context realism (i.e., how well they reflect real-world scenarios) and human-requirement realism (i.e., how accurately they capture user needs)^16^. Comparisons of current evaluation methods reveal that NLG assessments are often too narrow, falling short of the broader, human-centred approaches common in HCI^16^. Addressing this gap requires designing evaluations that better account for real-world contexts and human expectations.

Additional critiques emphasize the limitations of evaluating AI solely at the component level, advocating instead for system-level assessments. Barocas *et al*. distinguish between measuring individual components–such as the model or dataset–and evaluating the system as a whole. They argue that while component-level evaluations help identify the root causes of harm, system-level assessments are necessary to reveal performance disparities that directly relate to those harms^3^. Other scholars also stress the importance of extending fairness evaluations beyond the statistical modelling stage to include all stages of the machine learning (ML) pipeline, from data collection to deployment^20^.

This dataset supports the growing needs for systematic, rigorous, and comprehensive measurement practices for increasingly complex AI systems^21^, providing a foundation for investigating the aforementioned criticisms and enhancing measurement quality. It offers a consolidated resource for examining, comparing, and ultimately improving the quality of existing measures and their implementations. To create this resource, we conducted a scoping review^22^ to first examine how computing researchers operationalize ethical principles through measurement^23^. The review covered 257 papers published between 2011 to 2023 that claim to measure a normative construct of an AI component or system, for which many papers featured multiple measures. We then created a database summarizing essential information about each of these measures, framing the ethical principle as both a theoretical construct and a normative value under assessment, a full description of the measure and relevant criteria, the type of AI system and its application area, the specific AI system component measured, the type of assessment conducted, and the signalled sociotechnical harms by the measure. Features are organized under the major headings, including target outputs, entry points, connections to harm, measurement properties, algorithmic system characteristics, and publication metadata. Next, we processed the raw dataset by loading and reformatting it, cleaning inconsistencies, and computing descriptive and comparative statistics to summarize key patterns. To enhance readability and accessibility, we created an interactive visualization that allows users to explore the dataset.

The final dataset comprises over 12,067 data points spanning 791 measures, 11 principles, five AI system components, five types of assessments, nine application areas, and five sociotechnical harm types^21^.

## Methods

**Fig. 1:**
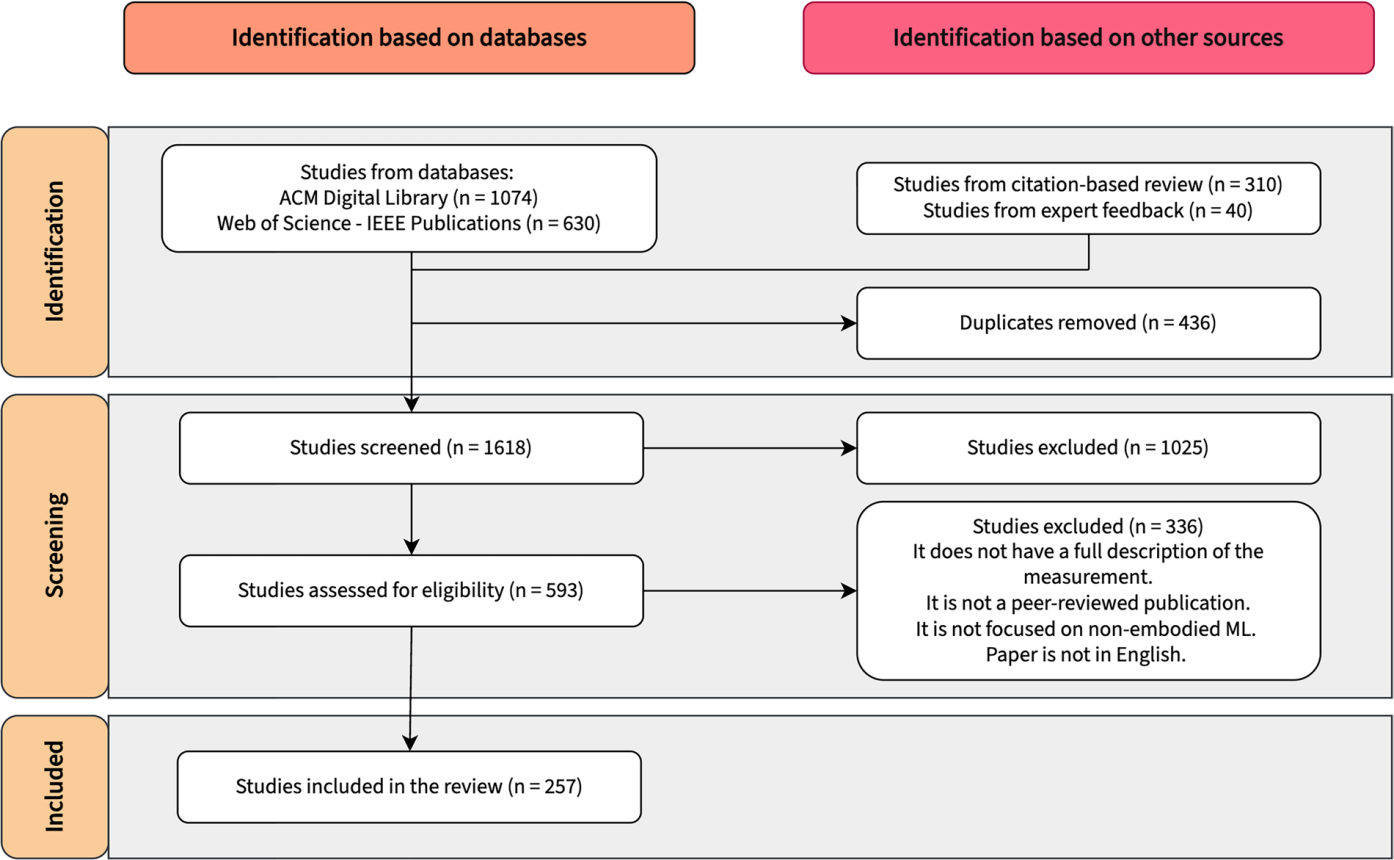
PRISMA flow diagram - The PRISMA flow diagram accounts for various stages in the scoping review process. The diagram illustrates the number of *papers* captured and screened in the review.

We conducted a scoping review to comprehensively identify and analyze existing measures for responsible AI development, focusing on the 11 ethical principles outlined by Jobin *et al*.^23^: fairness, transparency, trust, privacy, non-maleficence, beneficence, responsibility, freedom and autonomy, sustainability, solidarity, and dignity. Scoping reviews are well-suited for mapping research in emerging fields and providing structured overviews, making them an appropriate choice for this study^22,24,25^. We applied a structured five-stage scoping review approach: (1) identifying research questions; (2) identifying relevant studies; (3) study selection; (4) charting the data; and (5) collating, summarizing, and reporting results. We used Covidence (https://www.covidence.org/) to streamline the review process, ensuring consistent organization and documentation. The scoping review involved five reviewers with varying levels of expertise in measurements used for responsible AI development, providing oversight and rigour at each stage. The extraction process resulted in a dataset of 791 measures. Figure 1. summarizes the completed staged search strategy. After completing the review, we conducted a reflexive analysis to map the identified measures to specific types of sociotechnical harms. Finally, we refined the dataset to make it accessible and user-friendly for a broad audience and created an interactive visualization to support the navigation of the dataset^21^.

### Identify research questions

This scoping review focuses on answering the following research questions:

- **RQ1:** What measures and measurement processes are computing researchers proposing for assessing an AI system’s adherence to ethical principles?
- **RQ2:** What components of the AI systems are being measured when evaluating an AI system’s adherence to ethical principles?

We focused on ethical principles due to their critical role in responsible AI development and governance^26^, as well as their broad adoption across policy, academia, and industry^23^. Our focus on computing researchers stems from their central role in proposing measurement approaches to operationalize ethical principles and involvement in AI system development and implementation. This review specifically examines non-embodied AI systems, encompassing both standalone machine learning (ML) algorithms and ML-driven products.

### Identifying relevant studies

We adopted a staged search strategy^25^ to identify relevant resources and studies, including electronic scholarly databases, a citation-based review, and recommendations from domain experts.

#### Electronic scholarly databases

First, we conducted keyword searches in the Association for Computing Machinery (ACM) Digital Library (https://dl.acm.org/) and in Institute of Electrical and Electronics Engineers (IEEE) publications, which we accessed through the Web of Science (WoS) platform (https://clarivate.com/academia-government/scientific-and-academic-research/research-discovery-and-referencing/web-of-science/). Using WoS allowed for a more efficient extraction of paper titles and abstracts compared to directly scraping data from IEEE Xplore. We chose these peer-reviewed sources given their relevance to computing researchers and practitioners. We limited the search to papers published between 2017 and 2023 due to the growing popularity of the ethical principles research area during this period. For each of the 11 ethical principles, we constructed a set of search queries– on average about 10 per principle– based on three categories: (1) measures and synonymous terms (e.g., *evaluation* and *assessment)*; (2) AI, ML, and specific ML applications (e.g., *computer vision* (CV) and *natural language processing* (NLP)); and (3) each principle’s name and related codes identified by Jobin *et al*. For example, *justice* was used as a code for *fairness*.

We executed these queries across each database and documented the number of results obtained. Initially, full-text searches yielded up to 8000 results for more prevalent principles, such as fairness, with an overwhelming number of irrelevant papers. To refine the search, we restricted the queries to each article’s title, abstract, and keywords, ensuring search terms appeared within close proximity (3 to 5 words) of each other. For each query, we manually examined the abstracts and titles of the first 50 papers to assess contextual relevance and determine whether the paper proposed or used an ethical principle-related measure. In total, we tested 108 queries across the 11 principles, ultimately refining them to 22 final queries. Notably, the highest number of queries tested was for the principle of fairness, totalling 20 attempts, while responsibility had the lowest, with 5. The variation in the number of tested queries arises from the base query yielding more relevant results for some principles compared to others, which required more fine-tuning. The final list of queries and the resulting number of papers are included in Appendix A. The 22 queries yielded 1,074 papers from ACM Digital Library and 603 papers from IEEE. In total, 1,677 papers were passed on to the screening stage from the database search. We imported these into Covidence for a title and abstract screening and full-text review.

#### Citation-based review

To capture relevant literature potentially missed in the database searches, we conducted a citation-based review^27^ of the reference lists from papers that passed the full-text review and the review papers we captured from the database search. This allowed us to capture relevant literature not indexed within the electronic databases, including publications in major ML conferences and journal proceedings (i.e., Conference on Neural Information Processing Systems (NeurIPS), International Conference on Machine Learning (ICML), International Conference on Learning Representations (ICLR), Journal of Machine Learning Research (JMLR), and International Joint Conference on Artificial Intelligence (IJCAI)) and influential papers published between 2011 and 2016, earlier than the database search time frame. We chose this timeframe because of the rise of deep learning during this period, which was accompanied by the proposal of key measurements for ethical principles^28^. This review identified 310 papers (excluding duplicates)for screening.

#### Expert consultation

Upon completion of the citation-based review, we consulted three domain experts within our immediate network, who recommended an additional 40 papers (not accounting for the duplicates) for screening.

### Study selection

The study selection process involved multiple stages to ensure a comprehensive and structured screening of all relevant documents. We used Covidence to manage all stages, ensuring traceability and consistency. After removing 436 duplicates, 1618 papers passed on to title and abstract screening. Here, the titles and abstracts of all candidate documents were independently reviewed by three research assistants based on the inclusion and exclusion criteria (see Table 1). The lead project researcher trained all research assistants through a pilot title and abstract screening of 20 papers each. In the screening, papers receiving two “Yes” votes proceeded to full-text review, while those with two “No” votes were excluded. Conflicts were resolved by the lead project researcher. This process resulted in 593 papers for full-text review. These were distributed among two research assistants and the lead project researcher for independent review. The research assistants were again trained by completing ten full-text reviews with the lead project researcher. The research assistants flagged papers that they were uncertain about for subsequent review by the lead researcher. Justifications were documented for all exclusions. A total of 257 papers were included in the final corpus for data extraction. Figure 1 summarizes the Preferred Reporting Items for Systematic Reviews and Meta-Analyses (PRISMA) flow diagram for the scoping review process, documenting the number of papers at each stage and the reasons for exclusion.

**Table 1:** Inclusion and exclusion criteria used for article screening.

Inclusion Criteria	Exclusion Criteria
• Is the paper focusing on the design and development of a non-embodied ML system? • Is the paper proposing or applying a measure assessing adherence to one of the ethical principles identified in Jobin *et al*.? • *Database search*: Is the paper published between Jan 1st, 2017, and August 15th, 2023? • *Citation-based review:* Is the paper published between 2011 and 2016?	• The measure is for an embodied ML system (e.g., autonomous vehicles). • No measure is proposed or applied. • The measurement process is not fully described in the paper, and the measure is just mentioned. • The paper is published before 2011. • There is no mention of any of the ethical principles in the review paper by Jobin *et al*. • The publication is not peer-reviewed.

### Charting, collating, and summarizing of data

The data charting, collating, and summarizing process consisted of two main stages: data extraction and the conversion to individual measures.

#### Data extraction

We extracted data from the 257 papers using a predefined template in Covidence. This process was collaborative, involving two research assistants and the lead project researcher. For each paper, we extracted key elements, including:

- The ethical principle addressed (e.g., fairness, transparency, trust, sustainability);
- The measure and measurement process;
- The component of the AI system being assessed (e.g., input data, model, output, user interaction);
- The type of assessment (e.g., statistical, mathematical, self-reported, behavioural);
- The purpose of the ML system, data and algorithm types, and application areas;
- The article’s title, publication year, and key contributions.

Uncertainties were resolved through team discussion, which occasionally resulted in paper exclusions. Appendix B details the extraction template used.

#### Conversion to individual measures

**Fig. 2:**
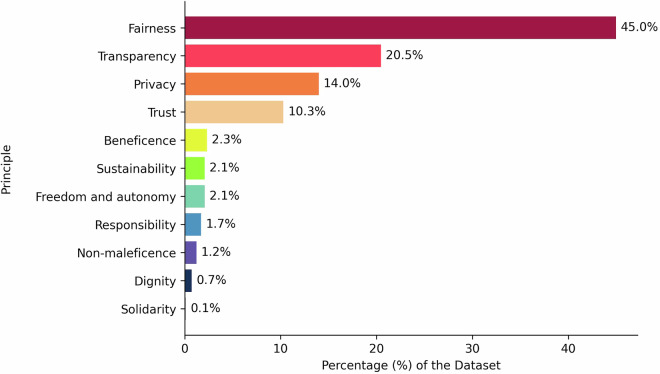
Distribution of measures across principles - A horizontal bar chart indicating the percentage of the measures across each of the eleven ethical AI principles in the dataset.

Many papers contained multiple measures, posing challenges for analysis and visualization. Using Microsoft Excel for greater flexibility in managing and categorizing data, we iteratively distinguished and isolated individual measures within each paper. First, a research assistant meticulously reviewed the dataset to identify and separate individual measures, resulting in a dataset comprising nearly 800 measures. Next, to improve the quality of the dataset, we refined the data extraction template, adding “criterion” and “criterion description” as new extractable elements, capturing the implicit or explicit criteria associated with each measure. To do this, a thorough review was then conducted by the lead project researcher and a senior researcher to ensure the quality of the extracted information and to add the new criterion data for each measure. Both researchers jointly reviewed 100 measures to check for consistency in how they finalized the measure and measurement process descriptions. The senior researcher then reviewed another 100 measures, with the lead project researcher reviewing the remaining measures. This iterative approach ensured data accuracy and clarity, enabling necessary analysis in the subsequent stages. The completed dataset contains 791 measures. Nearly half of the measures (45.0%) targeted fairness, followed by transparency (20.5%), privacy (14.0%), and trust (10.3%); these four principles yielded approximately 90% of all measures. Figure 2 illustrates the distribution of measures across the 11 ethical AI principles.

**Fig. 3:**
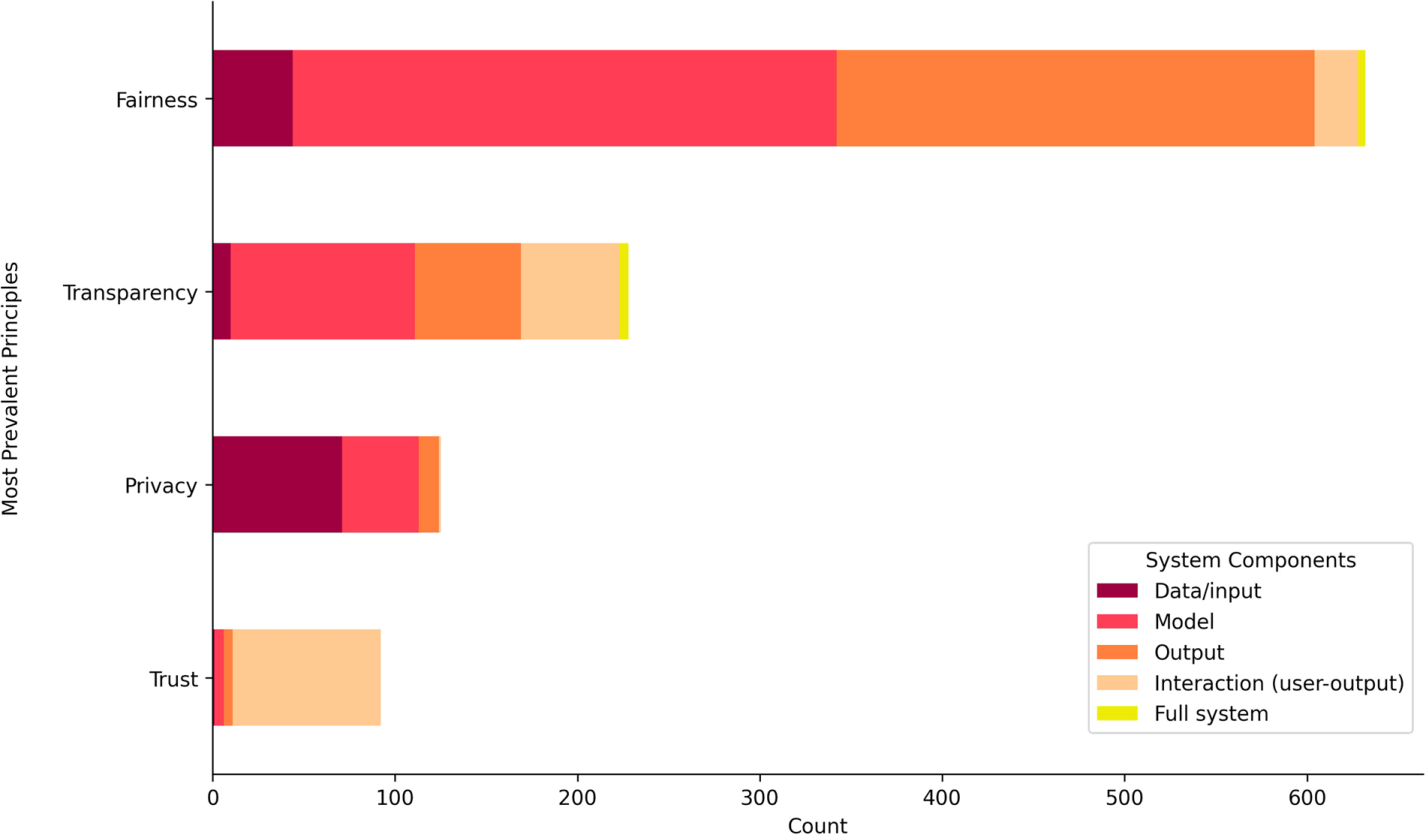
System components assessed for most prevalent principles - A horizontal stacked bar chart illustrating the relationship between measures associated with the four most prevalent responsible AI principles and the system components they assess.

**Fig. 4:**
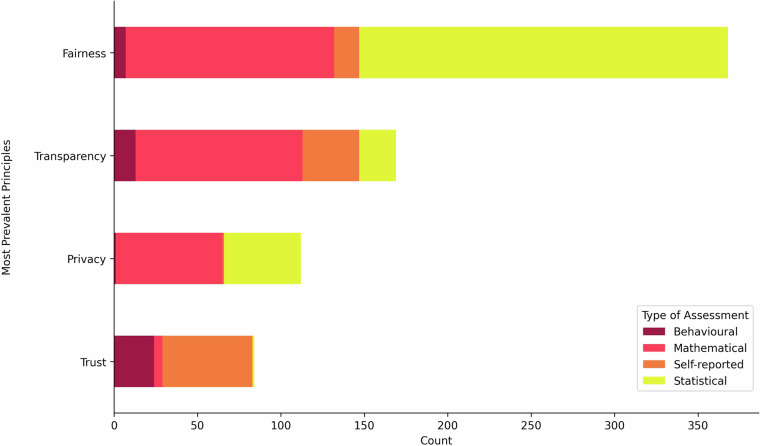
Assessment types linked to the most prevalent ethical AI principles - A horizontal stacked bar chart illustrating the relationship between measures associated with the four most prevalent ethical AI principles and the types of assessment conducted.

**Fig. 5:**
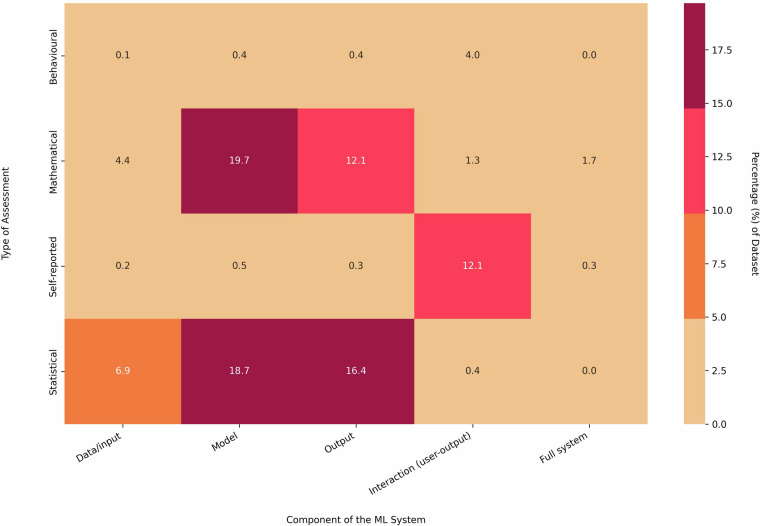
Assessment type by component - Heat map summarizing the relationship among system components and different assessment types.

From the four most prevalent principles, the distribution of the AI system components and types of assessments is explored in Figs. 3 and 4. Notably, for the principles of fairness and transparency, many of the measurements targeted the model and the output components of the AI system. Privacy measures predominantly focused on the input data and model components, whereas trust measures emphasized the user-output interaction (Fig. 3). Quantitative assessments, including mathematical and statistical evaluations, constituted the majority of measurements captured in the dataset, as reflected in Fig. 4 for the principles of fairness, transparency, and privacy^21^. In contrast, for the principle of trust, the distribution of assessment types differed, with a significant portion comprising behavioural and self-reported measures^21^. While comparing trends among the system components and types of assessments, the two most frequent types of assessment– mathematical and/or statistical– primarily targeted the model and output components^21^. Self-reported measures were more often linked to the user-output interaction component (Fig. 5).

### Connecting measures to a harm type

Given the importance of responsible AI measures in assessing the potential risks posed by AI systems, we conducted a reflexive analysis to examine which type(s) of sociotechnical harm each measure in our dataset signals^21^. We define sociotechnical harm as the adverse impacts that arise from the interplay between technical design decisions and broader social dynamics^29^. Specific manifestations of harms, such as discriminatory access to resources, stereotyping, or interpersonal manipulation, often serve as indicators of systemic risk^30^.

To assign a harm type to each measure, two researchers systematically reviewed the following information in the dataset: the measure itself, its measurement process, and key contextual details, such as the type of AI system, the criterion, and the paper's main contribution(s). When additional context was needed, we revisited the source paper. We used this information to determine the kind of sociotechnical harm(s) the measure aimed to signal. Harm types were categorized according to the five-part sociotechnical harm taxonomy introduced by Shelby *et al*.^29^: allocative harms, which involve economic or opportunity loss due to decisions assisted by or made by AI systems; representational harms, which reflect the reinforcement of unjust societal hierarchies or stereotypes; quality-of-service harms, which capture disparities in system performance across identity groups; interpersonal harms, which arise when AI systems adversely shape relationships between individuals or communities; and social system harms, which refer to broader, macro-level impacts of AI systems on societal structures or institutions.

To ensure consistency, two researchers independently coded a primary and, where applicable, secondary harm category for an initial set of 100 measures and discussed discrepancies to reach a consensus. The remaining measures were coded collaboratively, with the lead researcher handling most entries and consulting with the second researcher on complex cases to maintain consistency in classification. This harm-based categorization provides users of the dataset with an alternative way to explore the measures by mapping the types of sociotechnical harm(s) they aim to signal^21^.

### Data cleaning and processing

All computational analysis of the dataset was performed in a Jupyter Notebook (https://jupyter.org/) using Python (Version 3.13) with a CPU hardware accelerator. The data processing workflow consisted of four phases: loading the data; cleaning the data; computing descriptive and comparative statistics; and creating an interactive visualization tool for users to explore and engage with several key areas of the dataset.

#### Data loading

The raw data were loaded into the Jupyter Notebook environment.

#### Data cleaning

Next, several brief sanity checks were performed to ensure the raw data were properly carried over in a tabular format. Columns were renamed and reordered for clarity. No outliers exist in this data, though cells containing a significant excess of text from the extraction process were shortened for readability. Inconsistencies in case sensitivity, spacing and indentation, spelling, and punctuation formatting in each cell were handled using Pandas (https://pandas.pydata.org/) and NumPy (https://numpy.org/). All cells were carefully referenced to ensure no missing values were present. For the few existing empty cells, research assistants re-extracted the data from the source papers.

#### Descriptive and comparative statistics

After cleaning, we then calculated the counts, proportions, and percentages of measures associated with each of the eleven principles, and produced descriptive and comparative plots summarizing this information using the Matplotlib and Seaborn Python packages. Many papers and their corresponding measures provided coverage across multiple principles simultaneously. For instance, the principles of responsibility and non-maleficence both appear in Lewis *et al*.^31^, which uses the “Hellinger Distance” and “Total Variation Distance” measures for image classification. Additional checks were performed to cross-reference the raw data with the computed summary statistics for further verification; for instance, the counts were manually verified in the code and plots for several principles, system components, application areas, and types of assessments.

#### Interactive visualization

**Fig. 6:**
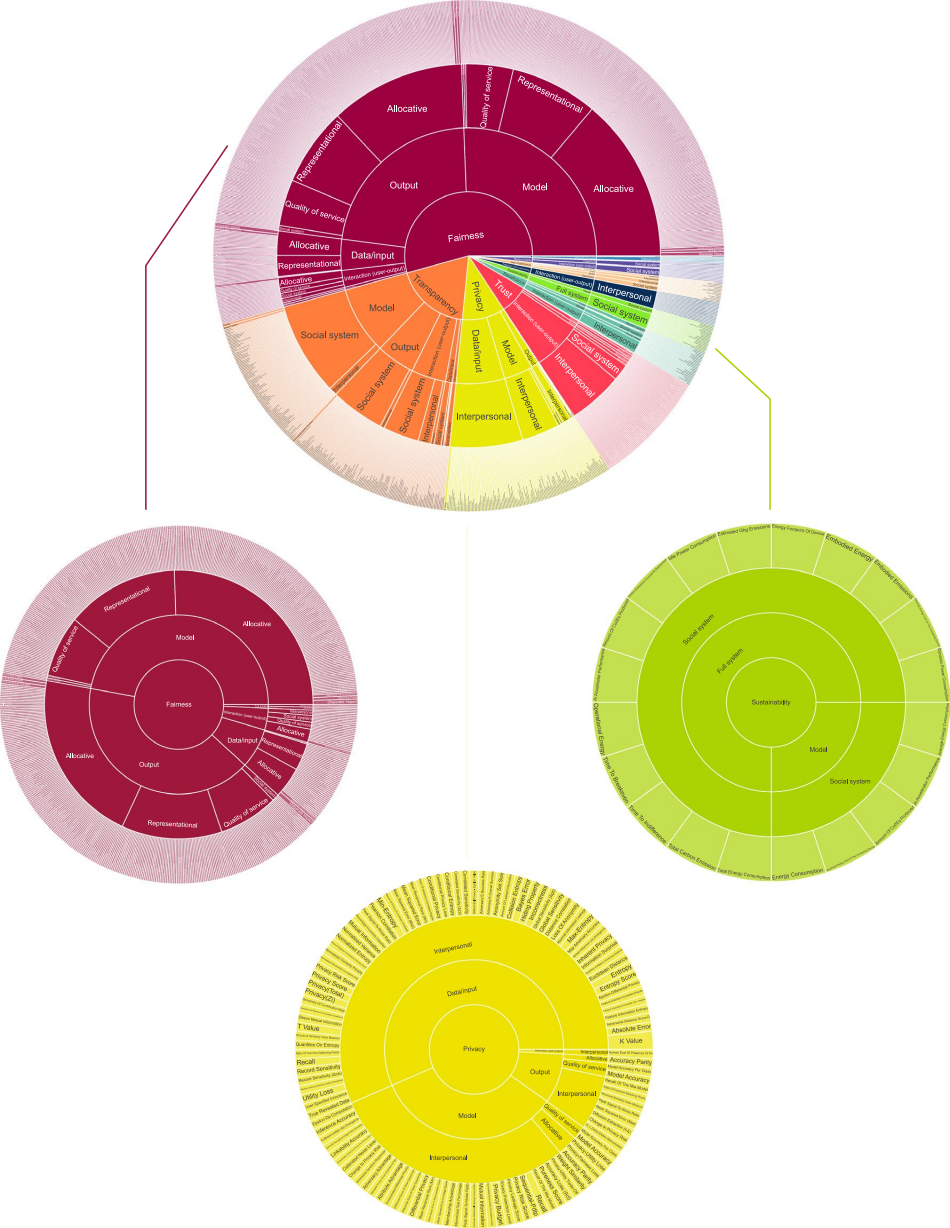
A Sunburst diagram detailing how the full visualization tool can be explored for each of the eleven principles; this figure shows the resulting measures of three of these principles: fairness, privacy, and sustainability.

We created pivot tables to examine the relationships between three columns: the principles, system components, and primary harm. We then generated a four-tier sunburst diagram to illustrate the hierarchical relationships between various parts of the dataset, primarily using the functionality from the Dash Python framework (https://pypi.org/project/dash/), built upon Plotly, React, and Flask. Render (https://render.com/), a web-server deployment platform, was used to host the visualization. The visualization details all eleven principles and their corresponding measures. Figure 6 provides an overview of the visual across three principle examples: fairness, privacy, and sustainability.

This visualization tool enables users to explore the dataset through interactive features including hover functionality and expandable sections in both forward and reverse passes, allowing for a customizable view of the structure and connections within the dataset^21^.

After all computational processing was completed, the resulting dataset contained 16 columns as summarized in Tables 2 and 3^21^. All variables are categorical or textual. To guide users of the dataset, we categorized each feature into one of six categories: target output, entry points, connections to harm, measurement properties, algorithmic system characteristics, and publication metadata. A summary description of these categorizations and dataset variables can be found in Table 2 for measurement-oriented dimensions and Table 3 for system and source-related context^21^.

**Table 2:** Overview of measurement-oriented variables in the Responsible AI measures dataset.

User Guidance Categories	Variable Name	Variable Type	Description
**Target Output**: The resulting measures collected in this dataset.	Measure	Textual	The name of the measure.
	Measurement Process	Textual	The measure description, including the evaluation process followed.
**Entry Points**: The primary features for filtering potential measures for an algorithmic system.	Principle	Categorical	The principle(s) are framed as the theoretical construct being measured in the paper (e.g., fairness, privacy, or sustainability). Eleven principles are considered.
	Part of the ML System	Categorical	The component of an AI system that is being assessed. Five categories are considered: input data, model, output, interaction with the user, and full system.
**Connections to Harm:** The associated sociotechnical harms with the measure.	Primary Harm	Categorical	The measure may relate to one of five possible sociotechnical harm types: allocative, representational, social system, interpersonal, and quality-of-service.
	Secondary Harm	Categorical	An optional column that may list a second sociotechnical harm.
**Measurement Properties:** The standard(s) used in each measure’s evaluation.	Criterion Name	Textual	The name of a standard by which something may be judged or decided in the measurement process.
	Criterion Description	Textual	A detailed description of the identified criterion.
	Type of Assessment	Categorical	Each measure can be described by at least one of five assessment types, including statistical, mathematical, behavioural, or self-reported.

**Table 3:** Overview of system and source related variables in the Responsible AI measures dataset.

User Guidance Categories	Variable Name	Variable Type	Description
**Algorithmic System Characteristics**: Additional features that a user can consider when narrowing down measures to use.	Application Area	Categorical	The general context in which this ML system is used (e.g., healthcare, education, or transportation).
	Purpose of ML System	Textual	The goal or objective of the ML system.
	Type of Data	Textual	The data format and type used in training and/or evaluating the ML system.
	Algorithm Type	Textual	The type of ML algorithm.
**Metadata:** Details further documentation into each source that was extracted to collect each feature and measure.	Title	Textual	The title of the paper containing each measure.
	Publication Year	Categorical	The publication year of the paper.
	DOI Link	Textual	Links to each publication and its corresponding measure(s).

## Data Records

All project files, including the dataset, are publicly available in a figshare repository^21^ containing five files: a README file, a raw Excel (.xlsx) dataset file, two code files, and another file with the website link to deploy the interactive visualization. The README file provides a detailed overview of the repository in a language accessible to any user, guiding them step-by-step. The raw data file, RAI_Measures_Dataset.xlsx, contains the data collected during the scoping review, organized into 16 columns as outlined in Table 2. There are two code files:the first is the Jupyter notebook code used to clean and process the data, as well as produce all plots, and the second file includes a Python script containing the visualization code and deployment protocol. The last file, “Sunburst_Visualization_Link.md” is a plain-text file linking users to the visualization, enabling them to explore the dataset interactively.

## Technical Validation

The technical validity of the Responsible AI Measures Dataset was ensured through meticulous adherence to established scoping review protocols^22,24,25^ to guarantee rigour and reliability. To achieve a comprehensive and unbiased collection of papers and measures, we implemented a systematic search, screening, full-text review, and data extraction process involving five reviewers. The team included research assistants who were thoroughly trained for their roles, while the two lead researchers with prior experience in conducting scoping reviews closely oversaw every stage to maintain quality and consistency. The reviewers brought diverse expertise spanning computer science, machine learning, software engineering, human-computer interaction, robotics, sociology, and science and technology studies, ensuring a multi-disciplinary perspective throughout the validation process. This rigorous approach allowed us to identify key trends in the dataset, which align with patterns reported in the existing literature^10,11^, further underscoring the reliability and broader relevance of our findings. Detailed checks were performed throughout the data collection and processing stages, ensuring that the cleaned dataset elements were verified against the raw data inputs.

## Usage Notes

### Guidance in file structure

A well-structured README file provides users with a detailed overview of the dataset’s content^21^. A hierarchical structure was also introduced within the dataset to help users differentiate between the 16 features (Table 2). These hierarchical headings include the purpose of each set of columns in the dataset:

- **Target Outputs:** The measures collected and their corresponding measurement process.
- **Entry Points:** Primary features that help narrow down the list of suitable measures for a user’s application.
- **Connections to Harm:** The sociotechnical harms that the measure aims to make aware and/or mitigate.
- **Further Criterion:** The standard(s) used in each measure’s evaluation.
- **Algorithmic System Characteristics:** Additional features that a user can consider while narrowing down measures to use.
- **Publication Metadata:** Further documentation on each source paper that was extracted to collect each feature and measure, including each article’s access link.

### Implementation of entry points and connections to harm

Both entry points, ethical principles and AI system components, and primary harm types detailed in Table 2, address the usability needs of stakeholders with different priorities, whether in regulation, development, infrastructure, or management. This approach aims to operationalize ethical principles within AI evaluation by engaging different priorities rather than emphasizing the importance of a single standpoint. The dataset features were specially collected to permit traceable, transparent, consistent, and most importantly, reflexive filtering, particularly within the visualization tool.

Users can navigate the dataset through six categorical variables: ethical principle, part of the AI system, primary and secondary harm types, type of assessment, and publication year^21^. Among these, the visualization currently supports exploration through three variables: ethical principle, part of the system, and the connections to primary harm types. First, each of the 11 ethical principles is marked as an entry point (e.g., fairness, transparency, trust, privacy, freedom and autonomy, responsibility, beneficence, non-maleficence, sustainability, dignity, and solidarity). Next, each of the five ML system components that this work has coded the measure to be contained within or related to (e.g., data/input, model, interaction (user-output), output, and the full system) is also noted as an entry point. Together, ethical principles and system components are labelled as the main entry points for navigating the dataset^21^. In the visualization, we also include primary harm type as a third entry point, which consists of five categories (e.g., representational, allocative, quality-of-service, interpersonal, and social system harms). Each of these variables provides a distinct way to navigate the dataset based on the potential needs of different users^21^. For example, practitioners may be interested in implementing or testing a specific principle within a pipeline component (e.g., goals of fairness in model training, transparency in the user interface, or privacy in the program’s outputs), while policymakers may be more interested in identifying gaps in the literature for certain principles or harm types across an AI system. Creating different entry points allows a wider range of users to access the data and make informed decisions based on their own context.

### Limitations, refinements and positionality

Despite the rigor of our methodology, the dataset has limitations. First, the field of responsible AI is rapidly evolving, with new measurements continuously emerging as we conducted the extraction process. While we aimed for comprehensiveness, it is possible that some recent contributions were not captured. To address this ongoing limitation, we will transform the dataset and its corresponding visualization tool into an open-source platform in future work, where researchers can contribute new measures. Second, our focus on computing research may underrepresent measures proposed by experts in policy, social sciences, and other disciplines, limiting the dataset’s scope across interdisciplinary boundaries. Third, while we aimed to distill key information, the dataset captures only specific elements from the papers; for a more nuanced understanding, readers may need to refer directly to the full text.

While we followed a meticulous and rigorous scoping review process with a diverse team and multiple quality checks, we acknowledge the inherent subjectivity in the selection and extraction process, as well as the influence of our positionality on the construction of this dataset. The authors are based in the United States and Canada, with diverse disciplinary backgrounds spanning computer science, machine learning, software engineering, human-computer interaction, robotics, sociology, and science and technology studies. With two members primarily from industry and four from academia, all authors have varying levels of experience in both sectors, which shaped our approach to data extraction and interpretation. For instance, inconsistencies in language or missing information in the reviewed papers required us to interpret and make decisions during data extraction. In addition, our familiarity with responsible AI measures commonly used in academia and industry informed our understanding. Lastly, we deliberately worked to represent how the papers themselves described each measure. These decisions were made with careful consideration to uphold the integrity of the dataset and ensure it reflects the diversity of the original sources.

## Supplementary information

Supplementary information

## Data Availability

The Responsible AI measures dataset is available as an Excel file at https://figshare.com/articles/dataset/_b_Responsible_Artificial_Intelligence_RAI_Measures_Dataset_b_/29551001?file=57701437^21^.

## Code availability

The project files are publicly available on the figshare repository, “Responsible_AI_Measures_Dataset” at the following location, https://figshare.com/articles/dataset/_b_Responsible_Artificial_Intelligence_RAI_Measures_Dataset_b_/29551001?file=57701437. Two code files have been included; the first file is Python code in a Jupyter notebook used to clean and process the data, in addition to producing all plots. The second code file is a Python script to create and deploy the interactive visualization. This repository is expected to remain active indefinitely. This dataset is published as Version 1.0. Updates will follow a major and minor versioning control scheme, with major additions indicated by the first number (e.g. 2.0 or 3.0), and minor changes indicated by the second number (e.g. 1.1 or 1.2). The README file will be updated to catalog all major and minor changes to the dataset, and all updates will be reflected on the figshare repository. We are currently working on a method to enable users to propose and contribute additional measures. This will allow the dataset to expand over time in a way that reflects emerging needs and uses. We are approaching this as a collaborative effort, consulting with practitioners and researchers from diverse backgrounds and disciplines to ensure the dataset remains relevant and inclusive across contexts.
