# Supplementary material for: Responsible AI measures dataset for ethics evaluation of AI systems
Source: Sci Data. 2025 Dec 20;12:1980. doi: 10.1038/s41597-025-06021-5 (PMC12722731; doi:10.1038/s41597-025-06021-5)
Supplement: Supplementary file 1 — Supplementary information [file 41597_2025_6021_MOESM1_ESM.pdf]

Supplementary information

Appendix A: Search Queries

Table S1. Search queries by ethical AI principle, number of papers retrieved, and query strings for ACM and IEEE (WoS)

**Principle**	**Total Number of Queries**	**Papers from ACM**	**Papers from IEEE (WoS)**	**Query for ACM**	**Query for IEEE (WoS)**
Transparency	9	154	123	[[Keywords: "machine learning"] OR [Keywords: "artificial intelligence"] OR [Keywords: "large language model"] OR [Keywords: "computer vision"] OR [Keywords: "generative models"] OR [Keywords: "natural language processing"] OR [Keywords: "predictive algorithm"]] AND [Abstract: transparency] AND [[Abstract: explain*] OR [Abstract: understand*] OR [Abstract: interpret*] OR [Abstract: disclos*] OR [Abstract: commun*] OR [Abstract: show*] OR [Abstract: explic*]] AND [[Abstract: evaluat*] OR [Abstract: metric] OR [Abstract: assess*] OR [Abstract: analy*]] AND [E-Publication Date: (01/01/2017 TO 08/31/2023)]	TS= (machine learning OR artificial intelligence OR large language model OR computer vision OR natural language processing OR predictive algorithm OR generative model OR recommender system) AND PUBL = (IEEE) AND AB = ((Transparency OR explainability OR explicability OR understandability OR interpretability) NEAR/7 (Metric OR Evaluat* OR Assess* OR Measur*))
Privacy	14	100	95	[[Keywords: "machine learning"] OR [Keywords: "artificial intelligence"] OR [Keywords: "large language model"] OR [Keywords: "computer vision"] OR [Keywords: "generative models"] OR [Keywords: "natural language processing"] OR [Keywords: "predictive algorithm"] OR [Keywords: "recommender system"]] AND [Keywords: privacy] AND [[Abstract: measur*] OR [Abstract: evaluat*] OR [Abstract: metric] OR [Abstract: assess*]] AND [[Abstract: privacy] OR [Abstract: "personal information"] OR [Abstract: or]] AND	(TS=(machine learning OR artificial intelligence OR large language model OR computer vision OR generative models OR natural language processing OR predictive algorithm OR recommender systems) AND PUBL=(IEEE)) AND AB=(privacy NEAR/3 (Measur* OR Metric OR Evaluat* OR Assess*))

				[[Abstract: privacy] OR [Abstract: "personal information"] OR [Abstract: "private information"]] AND [E-Publication Date: (01/01/2017 TO 08/31/2023)]	
Sustainability	8	25	40	[[Keywords: "machine learning"] OR [Keywords: "artificial intelligence"] OR [Keywords: "large language model"] OR [Keywords: "computer vision"] OR [Keywords: "generative models"] OR [Keywords: "natural language processing"] OR [Keywords: "predictive algorithm"] OR [Keywords: "recommender system"]] AND [[Abstract: analy*] OR [Abstract: evaluat*] OR [Abstract: metric] OR [Abstract: assess*]] AND [[Abstract: sustainability] OR [Abstract: energy] OR [Abstract: environment]] AND [Keywords: sustainability] AND [E-Publication Date: (01/01/2017 TO 08/31/2023)]	(TS=(machine learning OR artificial intelligence OR large language model OR computer vision OR generative models OR natural language processing OR predictive algorithm OR recommender system) AND PUBL=(IEEE) AND TS = sustainability) AND AB=((sustainability OR environment OR energy) NEAR/7 (Measur* OR Metric OR Evaluat* OR Assess* OR analy*))
Trust	10	55	96	[[Keywords: "machine learning"] OR [Keywords: "artificial intelligence"] OR [Keywords: "large language model"] OR [Keywords: "computer vision"] OR [Keywords: "generative models"] OR [Keywords: "natural language processing"] OR [Keywords: "predictive algorithm"]] AND [[Abstract: evaluat*] OR [Abstract: metric] OR [Abstract: assess*] OR [Abstract: measur*]] AND [Keywords: trust] AND [Abstract: trust] AND [E-Publication Date: (01/01/2017 TO 08/31/2023)]	(TS=(machine learning OR artificial intelligence OR large language model OR computer vision OR generative models OR natural language processing OR predictive algorithm OR recommender system) AND PUBL=(IEEE)) AND AB=(trust NEAR/3 (Measur* OR Metric OR Evaluat* OR Assess*))
Solidarity	11	86	15	[[Keywords: "machine learning"] OR [Keywords: "artificial intelligence"] OR [Keywords: "large language model"] OR [Keywords: "computer vision"] OR [Keywords: "natural language processing"] OR [Keywords: "predictive algorithm"] OR [Keywords: "generative model"] OR [Keywords: "recommender system"]] AND NOT [Keywords: "autonomous driving"] AND [[Full Text: solidarity] OR [Full Text: "impact on community"] OR [Full Text: "effect on community"] OR [Full	(TS=(machine learning OR artificial intelligence OR large language model OR computer vision OR generative models OR natural language processing OR predictive algorithm OR recommender system) AND PUBL=(IEEE)) AND AB=((solidarity OR "social impact" OR "social security" OR "cohesion")NEAR/7 (Measur* OR Metric OR Evaluat* OR Assess* OR analyz*))

				Text: "social impact"] OR [Full Text: "social security"] OR [Full Text: unity]] AND [[Keywords: evaluat*] OR [Keywords: metric] OR [Keywords: assess*] OR [Keywords: analy*] OR [Keywords: measure]] AND [E-Publication Date: (01/01/2017 TO 08/31/2023)]	
Autonomy and freedom	7	164	29	[[Keywords: "machine learning"] OR [Keywords: "artificial intelligence"] OR [Keywords: "large language model"] OR [Keywords: "computer vision"] OR [Keywords: "generative models"] OR [Keywords: "natural language processing"] OR [Keywords: "predictive algorithm"]] AND NOT [Keywords: "autonomous driving"] AND [[Abstract: freedom] OR [Abstract: autonomy] OR [Abstract: self-determination] OR [Abstract: liberty]] AND [[Abstract: evaluat*] OR [Abstract: metric] OR [Abstract: assess*] OR [Abstract: analy*] OR [Abstract: measur*]] AND [[Keywords: freedom] OR [Keywords: autonomy] OR [Keywords: self-determination] OR [Keywords: liberty]] AND [E-Publication Date: (01/01/2017 TO 08/31/2023)]	(TS=(machine learning OR artificial intelligence OR large language model OR computer vision OR generative models OR natural language processing OR predictive algorithm OR recommender system) AND PUBL=(IEEE)) AND AB=((freedom OR autonomy OR self-determination)NEAR/5 (Measur* OR Metric OR Evaluat* OR Assess* OR analyz*))
Responsibility	5	32	11	[[Keywords: "machine learning"] OR [Keywords: "artificial intelligence"] OR [Keywords: "large language model"] OR [Keywords: "computer vision"] OR [Keywords: "generative models"] OR [Keywords: "natural language processing"] OR [Keywords: "predictive algorithm"] OR [Keywords: or]] AND [[Abstract: analy*] OR [Abstract: evaluat*] OR [Abstract: metric]] AND [[Abstract: responsibility] OR [Abstract: accountability] OR [Abstract: "acting with integrity"]] AND [E-Publication Date: (01/01/2017 TO 08/31/2023)]	(TS=(machine learning OR artificial intelligence OR large language model OR computer vision OR generative models OR natural language processing OR predictive algorithm OR recommender system) AND PUBL=(IEEE)) AND AB=((responsibility OR accountability OR liability OR "acting with integrity") NEAR/5 (Measur* OR Metric OR Evaluat* OR Assess* OR analyz*))
Dignity	9	146	0	[[Keywords: "machine learning"] OR [Keywords: "artificial intelligence"] OR [Keywords: "large language model"] OR	(TS=(machine learning OR artificial intelligence OR large language model OR computer vision OR generative models OR natural

				[Keywords: "computer vision"] OR [Keywords: "generative models"] OR [Keywords: "natural language processing"] OR [Keywords: "predictive algorithm"] OR [Keywords: or]] AND [[Abstract: analy*] OR [Abstract: evaluat*] OR [Abstract: metric]] AND [[Abstract: responsibility] OR [Abstract: accountability] OR [Abstract: "acting with integrity"]] AND NOT [Abstract: transparency] AND [Abstract: quantitative] AND [E-Publication Date: (01/01/2017 TO 08/31/2023)]	language processing OR predictive algorithm OR recommender system) AND PUBL=(IEEE)) AND AB=((dignity OR "Self-respect" OR "Respectability") NEAR/5 (Measur* OR Metric OR Evaluat* OR Assess* OR analyz*))
Beneficence	9	14	24	[[Keywords: "machine learning"] OR [Keywords: "artificial intelligence"] OR [Keywords: "large language model"] OR [Keywords: "computer vision"] OR [Keywords: "generative models"] OR [Keywords: "natural language processing"] OR [Keywords: "predictive algorithm"] OR [Keywords: or]] AND [[Abstract: analy*] OR [Abstract: evaluat*] OR [Abstract: metric]] AND [[Abstract: responsibility] OR [Abstract: accountability] OR [Abstract: "acting with integrity"]] AND NOT [Abstract: transparency] AND [E-Publication Date: (01/01/2017 TO 08/31/2023)]	(TS=(machine learning OR artificial intelligence OR large language model OR computer vision OR generative models OR natural language processing OR predictive algorithm OR recommender system) AND PUBL=(IEEE)) AND AB=((beneficence OR well-being OR peace OR "social good" OR "common good") NEAR/5 (Measur* OR Metric OR Evaluat* OR Assess* OR analyz*))
Non-maleficence	6	127	26	[[Keywords: "machine learning"] OR [Keywords: "artificial intelligence"] OR [Keywords: "large language model"] OR [Keywords: "computer vision"] OR [Keywords: "generative models"] OR [Keywords: "natural language processing"] OR [Keywords: "predictive algorithm"] OR [Keywords: or]] AND [[Abstract: analy*] OR [Abstract: evaluat*] OR [Abstract: metric] OR [Abstract: measur*] OR [Abstract: assess*]] AND [[Abstract: responsibility] OR [Abstract: accountability] OR [Abstract: "acting with integrity"]] AND [[Keywords: responsibility] OR [Keywords: accountability]] AND [E-Publication Date: (01/01/2017 TO 08/31/2023)]	(TS=(machine learning OR artificial intelligence OR large language model OR computer vision OR generative models OR natural language processing OR predictive algorithm OR recommender system) AND PUBL=(IEEE)) AND AB=((non-maleficence OR safety OR harm) NEAR/3 (Measur* OR Metric OR Evaluat* OR Assess* OR analyz*))
Total	108	1074	630		

Appendix B: Original Data Extraction Template

I. General information

Title

Lead author

Year of publication

Key contributions

This section includes key contributions and the main purpose of the paper.

Notes

Include any important additional notes here

Principle and ML system information

Relevant principle (being measured)

Identify which principle is being assessed/measured in the paper. Make sure that you select the reference that the authors are saying is being measured.

- Fairness
- Transparency
- Trust
- Privacy
- Sustainability
- Solidarity
- Dignity

- Autonomy and freedom
- Responsibility
- Beneficence
- Non - maleficence

Purpose of machine learning system

In this field, describe the goal/objective of the machine learning system analyzed in the paper. An example response: a linear regression algorithm used to predict the likelihood of recidivism for criminals OR a transformer-based language model used for machine translation from German to English

Machine learning algorithm

Briefly identify the type of machine learning algorithm used in the paper and write 1-2 sentences describing it. Example: linear regression, support vector machine, transformer-based text to image model

Type of data

Describe the data type used for training and evaluating the ML model. write 1-2 sentences describing it. An example: tabular data about criminal history of a victim

Application area

Identify the general context in which this ML system is used

- Healthcare
- Education
- Transportation
- Journalism
- Criminal justice
- Other

II. Details of assessment

What part of the ML system is being assessed in this paper?

Identify what part of an ML system is being assessed. Feel free to use "other" box and the following question to fully explain what is being used.

- Data
- Model/algorithm
- Output
- Interaction between user and output
- Full ML system
- Other

If needed, elaborate on what part of the ML system is being assessed here.

What was the construct being measured (if different from the original principle)?

Name and write 1-2 sentences describing the construct. In most of the papers, the principle (Ex. fairness) will be the construct that is being assessed. However, in some scenarios, authors might have suggested other constructs that act as a proxy for the principle. For example, usability is often used as a construct for transparency.

What was the specific way of assessing/measuring the given construct?

This is where you will include the most amount of information about how the measurement is operationalized. This section can include formulas, steps, etc. As much as possible try to describe/give a brief summary of the formulas.

Type of assessment

Broadly categorize the type of assessment based on what you described in the previous questions. Statistical metrics use some statistical operation

on the data or the output. Self-reported questionnaires ask about user perception. Behavioural measures focus on the actions of the user. Qualitative studies could include general user observation or in-depth focus groups/interviews with them.

- Statistical metrics (choose if the metric involves statistics and probability)
- Mathematical representation (choose if metric is a mathematical interpretation of a given construct)
- Self-reported questionnaires
- Behavioural measures (choose if the metric is tracking human behaviour)
- Qualitative studies (observations, interviews)
- Other

If needed, elaborate on the type of assessment here. [Open box response]

III. Connection to harm and risk

How is the proposed assessment related to assessing magnitude of harm from the given ML system?

How is the proposed assessment giving any information about likelihood of harm from a given ML system?
